# Supplementary material for: Saliva as a testing specimen with or without pooling for SARS-CoV-2 detection by multiplex RT-PCR test
Source: PLoS One. 2021 Feb 23;16(2):e0243183. doi: 10.1371/journal.pone.0243183 (PMC7901781; doi:10.1371/journal.pone.0243183)
Supplement: S1 Table — (DOCX) [file pone.0243183.s001.docx]

S1 Table. Tentative LOD determination by series dilution*

*For each individual RT-PCR assay, a Ct value <40 indicates positive and a Ct>40 indicates negative. Accordingly, 100 copies/mL were determined as the tentative LOD.
