# Supplementary material for: Saliva as a testing specimen with or without pooling for SARS-CoV-2 detection by multiplex RT-PCR test
Source: PLoS One. 2021 Feb 23;16(2):e0243183. doi: 10.1371/journal.pone.0243183 (PMC7901781; doi:10.1371/journal.pone.0243183)
Supplement: S2 Table — a. Intra assay precision of the QuantiVirus SARS-Cov-2 test kit. b. Operator reproducibility of the QuantiVirus SARS Cov-2 test kit. c. Inter-instrument precision of the QuantiVirus SARS CoV-2 test kit. (DOCX) [file pone.0243183.s002.docx]

S2a Table. Intra assay precision of the QuantiVirus SARS-Cov-2 test kit

| **Target conc.** | **Orf1ab Gene (FAM)** | | | **N gene (CY5)** | | | **E gene (TexasRed)** | | | **Rp (IC)** | | |
| --- | --- | --- | --- | --- | --- | --- | --- | --- | --- | --- | --- | --- |
| **(copies/mL)** | **Mean Cq** | **% Replicate Detection** | **CV (%)** | **Mean Cq** | **% Replicate Detection** | **CV (%)** | **Mean**  **Cq** | **% Replicate Detection** | **CV (%)** | **Mean**  **Cq** | **CV (%)** |  |
| 100 | 33.76 | 100 | 0.67% | 35.97 | 100 | 2.85% | 37.87 | 100 | 1.52% | 29.68 | 0.67% |  |
| 200 | 32.90 | 100 | 1.76% | 35.35 | 100 | 2.22% | 37.31 | 100 | 2.26% | 29.62 | 1.76% |  |
| 300 | 32.28 | 100 | 0.43% | 34.64 | 100 | 1.27% | 36.42 | 100 | 2.44% | 29.87 | 0.43% |  |
| 500 | 31.42 | 100 | 1.50% | 33.59 | 100 | 0.87% | 35.53 | 100 | 1.23% | 30.18 | 1.50% |  |

S2b Table. Operator reproducibility of the QuantiVirus SARS Cov-2 test kit

S2c Table. Inter-instrument precision of the QuantiVirus SARS CoV-2 test kit **
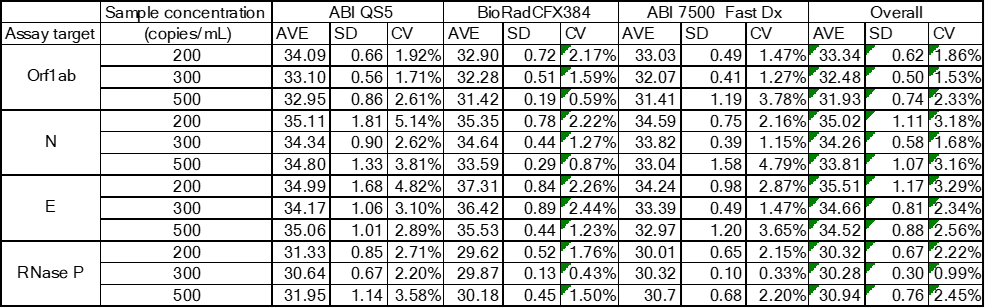
**
