# Supplementary material for: Saliva as a testing specimen with or without pooling for SARS-CoV-2 detection by multiplex RT-PCR test
Source: PLoS One. 2021 Feb 23;16(2):e0243183. doi: 10.1371/journal.pone.0243183 (PMC7901781; doi:10.1371/journal.pone.0243183)
Supplement: S3 Table — (DOCX) [file pone.0243183.s003.docx]

S3 Table. Results of cross-reactivity evaluation of the QuantiVirus ^TM^ SARS CoV-2 test kit

*RP, internal control; NTC, no target control; PC-kit, kit positive control; PC_4gblock, gblock contains all target gene positive controls**.**
