# Supplementary material for: Saliva as a testing specimen with or without pooling for SARS-CoV-2 detection by multiplex RT-PCR test
Source: PLoS One. 2021 Feb 23;16(2):e0243183. doi: 10.1371/journal.pone.0243183 (PMC7901781; doi:10.1371/journal.pone.0243183)
Supplement: S4 Table — a. Evaluation of contrived saliva samples with added non-infectious viral particles (Bio-Rad CFX 384). b. Evaluation of contrived saliva samples with added non-infectious viral particles (ABI QuantStudio 5). c. Evaluation of contrived saliva samples with added non-infectious viral particles (ABI 7500 Fast Dx). (DOCX) [file pone.0243183.s004.docx]

S4a Table. Evaluation of contrived saliva samples with added non-infectious viral particles (Bio-Rad CFX 384)

 *Note: Contrived saliva samples include 40 positives and 30 negatives.

S4b Table. Evaluation of contrived saliva samples with added non-infectious viral particles (ABI QuantStudio 5)

S4c Table. Evaluation of contrived saliva samples with added non-infectious viral particles (ABI 7500 Fast Dx)

*Three tables data were Republished from [Ref 14, FDA EUA] under a CC BY license, with permission from [Diacarta Inc], original copyright [2020].
