# Supplementary material for: Saliva as a testing specimen with or without pooling for SARS-CoV-2 detection by multiplex RT-PCR test
Source: PLoS One. 2021 Feb 23;16(2):e0243183. doi: 10.1371/journal.pone.0243183 (PMC7901781; doi:10.1371/journal.pone.0243183)
Supplement: S5 Table — (DOCX) [file pone.0243183.s005.docx]

S5. Table. Paired NPS and saliva samples tested by QuantiVirus ^TM^ SARS-CoV-2 test
