# Supplementary material for: Saliva as a testing specimen with or without pooling for SARS-CoV-2 detection by multiplex RT-PCR test
Source: PLoS One. 2021 Feb 23;16(2):e0243183. doi: 10.1371/journal.pone.0243183 (PMC7901781; doi:10.1371/journal.pone.0243183)
Supplement: S6 Table — (DOCX) [file pone.0243183.s006.docx]

S6 Table. Assay sensitivity evaluation of various FDA EUA approved SARS-CoV-2 RT-PCR test Kits *

*Update on Jan 9, 2021. Ten kits were selected here for comparison. The FDA SARS-CoV-2 Reference Panel allows for a more precise comparison of the analytical performance of different molecular in vitro diagnostic (IVD) assays intended to detect SARS-CoV-2. The Reference Panel contains common, independent, and well-characterized reference material that is available to developers of SARS-CoV-2 nucleic acid-based amplification tests (NAATs) for which Emergency Use Authorization (EUA) was requested. <https://www.fda.gov/medical-devices/coronavirus-covid-19-and-medical-devices/sars-cov-2-reference-panel-comparative-data>
